# Short-Time Antibacterial Effects of Dimethylaminododecyl Methacrylate on Oral Multispecies Biofilm In Vitro

**DOI:** 10.1155/2019/6393470

**Published:** 2019-01-21

**Authors:** Yujie Zhou, Suping Wang, Xuedong Zhou, Yiran Zou, Mingyun Li, Xian Peng, Biao Ren, Hockin H. K. Xu, Michael D. Weir, Lei Cheng, Yu Chen, Qi Han

**Affiliations:** ^1^State Key Laboratory of Oral Diseases & National Clinical Research Center for Oral Diseases & Department of Cariology and Endodontics & Department of Oral Pathology, West China Hospital of Stomatology, Sichuan University, Chengdu 610041, China; ^2^Department of Operative Dentistry and Endodontics & Stomatology Center, The First Affiliated Hospital of Zhengzhou University, Zhengzhou 450052, Henan, China; ^3^Department of Advanced Oral Sciences and Therapeutics, University of Maryland Dental School, Baltimore, MD 21201, USA

## Abstract

Quaternary ammonium compounds constitute a large group of antibacterial chemicals with a potential for inhibiting dental plaque. The aims of this study were to evaluate short-time antibacterial and regulating effects of dimethylaminododecyl methacrylate (DMADDM) on multispecies biofilm viability, reformation, and bacterial composition in vitro. DMADDM, chlorhexidine (CHX), and sodium fluoride (NaF) were chosen in the present study.* Streptococcus mutans*,* Streptococcus sanguinis,* and* Streptococcus gordonii *were used to form multispecies biofilm. Cytotoxicity assay was used to determine the optimal tested concentration. 3-(4,5-dimethyl-thiazol-2-yl)-2,5-diphenyltetrazolium bromide (MTT) assay and resazurin test of biofilm were conducted to study the biomass changes and metabolic changes of controlled multispecies biofilm. Scanning electron microscopy (SEM) was used to observe biofilm images. TaqMan real-time polymerase chain reaction was performed to evaluate the proportion change in multispecies biofilm of different groups. Cytotoxicity assay showed that there existed a certain concentration application range for DMADDM, CHX, and NaF. MTT assay and resazurin test results showed that DMADDM and CHX groups decreased multispecies biofilm growth and metabolic activity (*p *< 0.05), no matter after 1 min or 5 min direct contact killing or after 24 h regrowth. The proportion of* S. mutans* decreased steadily in DMADDM and CHX groups after 1 min and 5 min direct contact killing and 24 h regrowth, compared to control groups. A novel DMADDM-containing solution was developed, achieving effective short-time antibacterial effects and regulation ability of biofilm formation.

## 1. Introduction

The human mouth is home to the second biggest microbial community in the body, with more than 700 species of bacteria residing on the hard surfaces of teeth and soft tissues of oral mucosa, which exist in the form of micro-biofilms [1–3]. As the most complex and diverse microbial communities in the human body, the balance of oral microbiome is essential for maintenance of host oral and body health [4, 5]. Once disrupted, imbalanced oral microbiome could cause a variety of oral infections, e.g., dental caries, gingivitis, and oral mucositis (refers to an inflammatory, erosive and/or ulcerative process of oral mucosa) [6, 7]. For practitioners and patients alike, effectively maintaining oral microbiome balance is therefore important to promote or restore human oral health.

Using chemical antiseptics is one of the main strategies for suppression of oral microbial biofilm [8, 9]. For example, the application of antimicrobial oral rinses (mouthwashes) plays an important role in controlling dental plaque and gingivitis or maintaining oral hygiene [10–12]. Mouthwashes containing chlorhexidine (CHX) is the most representative, with their efficacy in reducing oral bacterial viability, inhibiting plaque regrowth, and preventing gingivitis, which has been demonstrated in many studies [13–16]. Although microbial plaques biomass was reduced after oral antiseptic treatments, the influence of antibacterial agents on interindividual variations of oral biofilm remains to be known. The pathogenicity of the oral biofilm is related to its properties, which is depending on the internal biological micro-composition [17, 18]. The composition of the bacterial community and its spatial distribution have been studied in various ways to reveal a highly structured organization of biofilm [18]. Thus, how to effectively adjust micro-ecological balance is of great importance. By now, seldom studies focused on the microbial composition changes after biofilm exposure to antibacterial agents or on the connection between microbial composition and antibiofilm properties of antibacterial agents.

Quaternary ammonium methacrylates (QAMs) were identified to be effective against dental biofilms and promising for dental clinical applications [19]. The positively charged quaternary amine N^+^ of QAMs could interact with the negatively charged cell membrane of the bacteria, which could cause membrane disruption and cytoplasmic leakage, leading to bacterial death [20]. In previous studies, many different kinds of QAMs, e.g., 12-methacryloyloxy-dodecyl-pyridinium bromide (MDPB), nanoparticles of quaternary ammonium polyethylenimine (QPEI), were synthesized and applied into a variety of dental polymeric materials, e.g., resin composites, adhesives, via formation of chemical bonds between dental resin matrix monomers, which plays a nonreleasing antimicrobial efficacy [21–25]. However, there is no study exploring the relationship between different concentrations of QAMs solutions and their micro-ecological regulation function. Dimethylaminododecyl methacrylate (DMADDM) is a member of QAMs with better antibacterial performance, which has a 12 alkyl chain length (CL=12) [23]. Previous studies have found that DMADDM had strong antibiofilm effects on growth of planktonic bacteria and inhibition of bacterial adhesion [23–27]. However, no study investigated the effects of DMADDM-containing solution on multispecies biofilm and evaluated the composition changes of multispecies biofilm after short-time killing treatment.

Therefore, the objectives of this study were to (1) compare the antibacterial effects of aqueous solution of DMADDM, CHX, and sodium fluoride (NaF) on oral biofilm growth and regrowth process after short-time killing treatments and (2) investigate their regulation effects on micro-biofilm composition in vitro.

## 2. Materials and Methods

### 2.1. Synthesis of QAMs with Long Alkyl Chain Length

DMADDM was synthesized via a modified Menschutkin reaction method [28]. To synthesize DMADDM, 10 mmol of 2-bromoethyl methacrylate (BEMA, Portland, OR, USA), 10 mmol of 1-(dimethylamino)dodecan (DMAD, Portland, OR, USA), and 3 g of ethanol were added to a vial, which was capped and stirred at 70°C for 24 h. After the reaction was completed, ethanol was evaporated. Then DMADDM was obtained as a clear liquid, which was verified via Fourier transform infrared spectroscopy (FTIR).

### 2.2. Cell Culture

Experiments were carried out using human buccal epithelial cells (HBECs, TR146 cells, JENNIO Biological Technology, Guangzhou, China). TR146 cells were routinely cultured in Dulbecco's Modified Eagle's Medium (DMEM, HYclone) supplemented with 10% fetal bovine serum (FBS, Gibco, USA) and 1% penicillin-streptomycin. All experiments were carried out in serum-free DMEM.

### 2.3. Cytotoxicity Assays

TR146 cells were grown to confluence on 96-well plates for 24 h in DMEM medium. DMADDM solution was added to DMEM medium with 100 *μ*L/well and incubated for 1 min or 5 min (37°C, 5% CO_2_) before being moved by aspiration. After washing by 3 mL of phosphate buffered saline (PBS), cells were soaked into 100 *μ*L/well DMEM with 10 *μ*L of the Cell Counting Kit-8 (CCK-8) solution at 37°C for 2 h. The suspension was used to measure absorbance at 490 nm using a microplate reader (SpectraMax M5, Molecular Devices, USA). For negative cell control, TR146 cells were incubated in DMEM without DMADDM. For positive cells control, 1% Triton X-100 was added during the coculture. For background control, there were no cells in the wells and DMEM with the CCK-8 solution was used for detection.

### 2.4. Microbial Cultures and Multispecies Biofilm Formation

*S. mutans *(UA159),* S. sanguinis* (ATCC 10556), and* S. gordonii* (ATCC 10558) were all supplied by the State Key Laboratory of Oral Diseases (Sichuan University, Chengdu, China).* S. mutans*,* S. sanguinis,* and* S. gordonii* were routinely cultured in brain heart infusion broth (BHI, Oxoid, Basingstoke, UK) at 37°C anaerobically (5% CO_2_, 90% N_2_, 5% H_2_) for 12 h for preparing multispecies biofilm formation. For multispecies biofilm formation, bacterial routine suspensions were mixed to obtain an inoculum containing a multispecies microbial population consisting of* S. mutans* (10^7^ (Colony forming unit, CFU) /mL),* S. sanguinis* (10^7^ CFU/mL), and* S. gordonii* (10^7^ CFU/mL) in 4% BHI with 1% sucrose. The medium used for biofilm development was exchanged every 12 h.

Hydroxyapatite (HA) disks were all supplied by the National Engineering Research Center for Biomaterials (Sichuan University, Chengdu, China). Triple-A model was used to form multispecies biofilm [29]. The model consists of a custom-made stainless steel lid with 24 clamps. Each clamp holds one hydroxyapatite disc vertically. The clamps were positioned to allow inserted hydroxyapatite disk fit into the wells of a 24-well tissue culture plate. The lid assembled with clamps and hydroxyapatite disk were sterilized in an ethylene oxide sterilizer (AnproleneAN 74i, Andersen, Haw River, NC, USA) before use. The final coculture for biofilm formation was composed of each bacteria with 10^7^ of colony forming unit (CFU)/mL in 1.6 mL/well BHI medium with 1% sucrose in 24-well plates with vertical HA disks placed at 37°C aerobically for 48 h and the media was refreshed every 12 h before test.

### 2.5. Antiseptics Killing the Multispecies Biofilm

After 48 h incubation, the specimens were transferred to two new 24-well plates with 1.5 mL of each antiseptic; one was maintained in contact for 60 s and another for 5 min. The tested antiseptics were 200 *μ*g/mL and 40 *μ*g/mL of DMADDM, 0.2% and 0.01% of CHX, and 0.05% of NaF. Demineralized water was used for the control group. The biofilm-related tests were performed after contacting the antimicrobial agent immediately or after exposure to the antimicrobial agent for 24 h, which were expressed as “1 min/ 5 min-right after killing” and “1 min/ 5 min-right after 24 h”, respectively.

### 2.6. MTT Assay

The 3-(4,5-dimethyl-thiazol-2-yl)-2,5-diphenyltetrazolium bromide (MTT) assay is a colorimetric assay for assessing three-species metabolic activity, which measures the enzymatic reduction of MTT (a yellow tetrazole) to formazan and is used to test the bacterial viability and bacterial reproductive capacity of biofilm on the disks. Disks with 48 h biofilm after killing were rinsed with demineralized water for 3 times to remove loose bacteria and residual antiseptics. Each disk was transferred to a new 24-well plate, to which the MTT solution (0.5 mg/mL MTT in PBS) was added and incubated at 37°C in 5% CO_2_ for 1 h. Metabolically active bacteria metabolized the MTT and reduced it to purple formazan inside the living cells during this 1 h. After that, the disks were transferred to a new 24-well plate, 1 mL of dimethyl sulfoxide (DMSO, Sigma, USA) was added to solubilize the formazan crystals, and the plate was incubated for 20 min with gentle mixing at room temperature. After mixing via pipetting, 200 *μ*L of the DMSO solution from each well was transferred to a new 96-well plate. The absorbance was monitored at absorbance of 540 nm (OD_540 nm_) with the microplate reader (SpectraMax M5, USA). Six replicates were tested for each group.

### 2.7. Resazurin Tests of Biofilm

Resazurin (7-hydroxy-3H-phenoxazine-3 one 10-oxide), referred to as Cell Titer-Blue (CTB) or Alamar Blue, is stable and nontoxic. Biofilm on disks after killing treatment was cleaned with deionized water, then we transferred them into 1.5 mL DMEM medium without phenol red with 0.0016% resazurin (Sigma-Aldrich, USA). After 90 min at 37°C anaerobically (5% CO_2_, 90% N_2_, 5% H_2_), the disks were transferred to a new 24-well with 1.5 mL new BHI medium. The used medium with 0.4% glucose transferred 100 *μ*L to 96 well plate for test. The measurement of the absorbance (Excitation: 485 nm, emission: 580 nm) for the medium with 0.4% glucose was read through the microplate reader (SpectraMax M5, USA).

### 2.8. Scanning Electron Microscopy Observation

Scanning electron microscopy (SEM) was used to examine structural and morphological changes of the biofilm. For SEM images, the biofilm on HA disks was rinsed with PBS and then immersed in 1% glutaraldehyde for 4 h at 4°C. Then, the specimens were subjected to graded ethanol dehydrations and rinsed twice with 100% hexamethyldisilazane. The specimens were then sputter-coated with gold and examined using SEM (Quanta 200, FEI, Hillsboro, OR, USA).

### 2.9. DNA Isolation and Real-Time Polymerase Chain Reaction

Total DNA of biofilm was isolated and purified using a TIA Namp Bacteria DNA kit (TIANGEN, Beijing, China) according to the manufacturer's instructions. The bacteria were lysed using enzymatic lysis buffer (20 mM Tris-HCl, pH 8.0; 2 mM sodium EDTA and 1.2% Triton X-100) containing 25 mg/mL of lysozyme at 37°C for 1.5 h. The purity and concentration of DNA were detected by NanoDrop 2000 spectrophotometer (Thermo Scientific, MA, USA). The extracts were stored at −20°C for later use.

TaqMan real-time polymerase chain reaction (PCR) was performed for the quantitative detection of* S. mutans*,* S. sanguinis,* and* S. gordonii* by using a Bio-Rad CFX96 TM Real-time System (Bio-Rad, CA, USA). Each real-time PCR reaction mix containing 10 *μ*L of TaqMan Universal PCR Premix Ex Taq, 1.5 *μ*L of template, 250 nM (each) of sense and antisense primer, and 250 nM of TaqMan probe was placed into each well, and the cycling conditions used are as follows: 95°C for 3 min, followed by 40 cycles of 95°C for 10 s and 56°C for 30 s. Fluorescence was detected after each cycle. The specificity of probes was confirmed by conventional PCR, and the standard curves of these bacteria were plotted for each primer/probe set by using threshold cycle values obtained by amplifying successive 10-fold dilutions of known concentrations of DNA which stands for the corresponding concentration of bacteria from 10^9^ CFUs to 10^4^ CFUs. The quantifications of three strains were calculated based on standard curves generated using respective standard strains. Three replicates were tested for each group.

### 2.10. Statistical Analysis

One-way ANOVA and Mann–Whitney U-test was used to compare the resazurin assay and MTT test. Statistical analysis was conducted with Mann–Whitney U-test to detect the significant effects of the variables. For this experiment, the independent variables referred to different treatment groups of antibacterial agents and the dependent variables referred to multispecies biofilm before any treatment. Statistical analyses were performed by SPSS 21.0 software (SPSS, Chicago, IL) at p < 0.05.

## 3. Results

### 3.1. Cell Cytotoxicity Test

**Figure 1:**
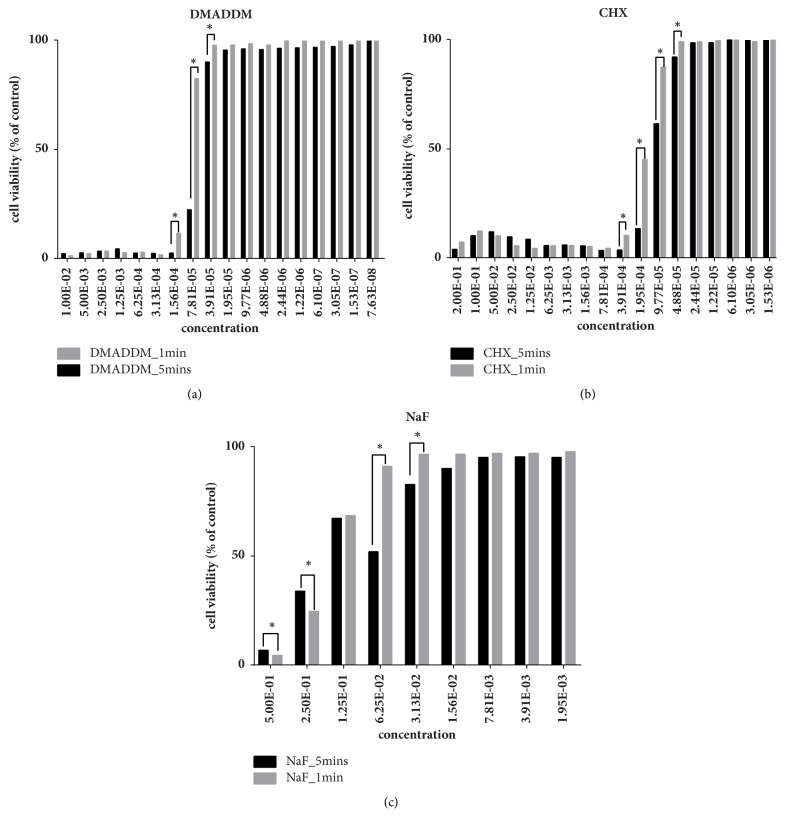
Cell viability tests of human buccal epithelial cells after being exposed to DMADDM (a) or CHX (b) or NaF (c) for 1 min or 5 min.

The concentration of antimicrobial agent in this experiment was screened by cytotoxicity experiment.  Figure 1 showed the results of CCK-8 assay, the absorbance rates decreased with increasing concentrations and incubation times based on negative values observed in negative control group. For DMADDM (Figure 1(a)), significant toxicity was observed at concentration of 200 *μ*g/mL, with the mean cell viability rate of less than 11.53% after 1 min treatment. However, at concentration of 78.1 *μ*g/mL and lower concentration groups, no significant toxicity was detected. 5 min test indicated that below 40 *μ*g/mL DMADDM was safe to HBECs line even after incubation, with the mean cell viability rate of 90.22%. CHX is considered the gold standard against gram-negative microorganisms, and CHX is widely used in clinic as a major mouthwash formulation product. Therefore, groups of CHX were chosen as our positive control to evaluate the cell toxicity (Figure 1(b)). No significant toxicity was observed at concentrations of up to 0.01% CHX in contrast to the lower groups, with the cell viability rate of 87.54% after 1 min incubation. For 5 min, group of 0.005% CHX showed cell viability rate of 92.20%, which had no significance with lower groups (Figure 1(b)). As for NaF, no significant toxicity was observed at concentrations of up to 6.25% NaF in contrast to the groups of lower concentration, with the cell viability rate of 91.24% for 6.25% NaF after 1 min incubation (Figure 1(c)), and no significant toxicity was observed at concentrations of up to 1.56% NaF in contrast to the groups of lower concentration, with the cell viability rate of 90.21% for 1.56% NaF after 5 min incubation.

Furthermore, based on the results of the CCK-8 assay, high and low concentration tested antibacterial agent groups were selected for our study as follows: 200 *μ*g/mL DMADDM (D_H), 40 *μ*g/mL DMADDM (D_L), 0.2% CHX (CHX_H), 0.01% CHX (CHX_L), 1.56% NaF (NaF), respectively.

### 3.2. MTT Assay

**Figure 2:**
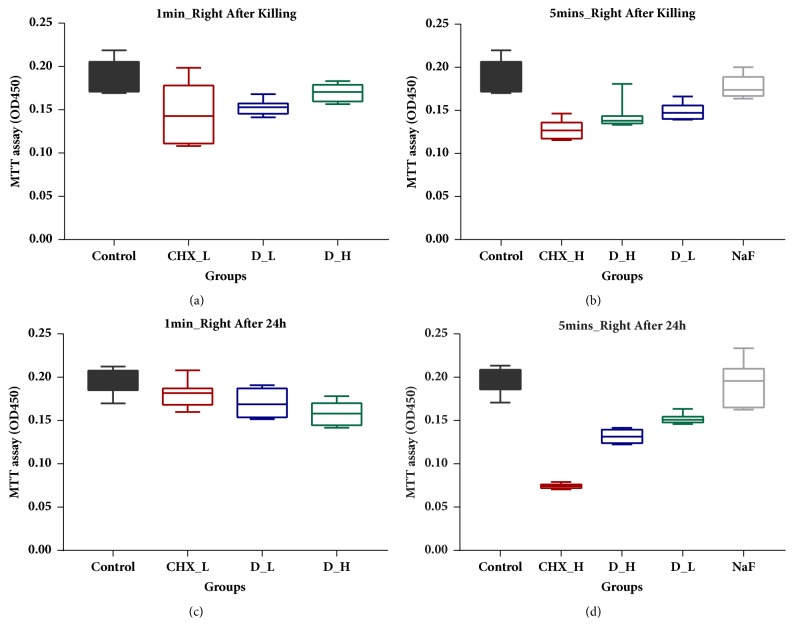
MTT assay. (a) Absorbance values of biofilm after 1 min killing treatment. (b) Absorbance values of biofilm after 5 min killing treatment. (c) Absorbance values of regrowth 24 h biofilm after 1 min killing treatment. (d) Absorbance values of regrowth 24 h biofilm after 5 min killing treatment.

Cell proliferation effects were investigated using MTT assay, as shown in Figure 2. Compared with control group (demineralized water), all concentrations of DMADDM and CHX caused a decrease in absorbance rates of biofilm biomass at tested time points (Figure 2), while 0.2% CHX exerted the most significant impacts on cell proliferation, with least regrowth of bacterial biomass after 24 h (Figure 2(d)). Similarly, cell proliferation was detected in the DMADDM groups, with a slight increase in the absorbance rates at time points of 1 min and 5 min, which indicated that DMADDM was efficient in killing bacterial biomass (Figures 2(a) and 2(b)). The same situation occurred in DMADDM groups after 24 h biofilm regrowth (Figures 2(c) and 2(d)), suggesting its effective long-term antibacterial activity. As for NaF, no significant difference of cell proliferation was observed between NaF and control group for 5 min killing (Figures 2(b) and 2(d)).

### 3.3. Resazurin Metabolism Assay

**Figure 3:**
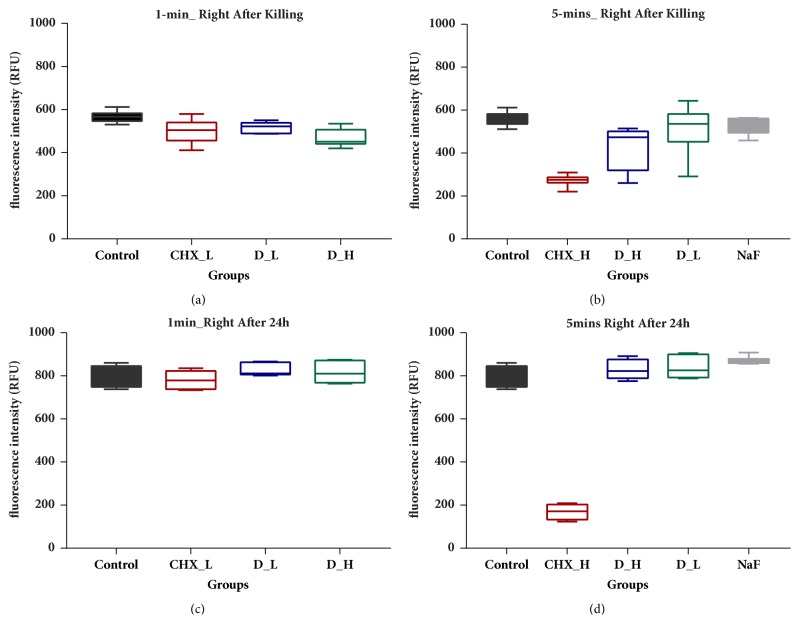
Resazurin metabolism assay. (a) Fluorescence intensity of biofilm after 1 min killing treatment. (b) Fluorescence intensity of biofilm after 5 min killing treatment. (c) Fluorescence intensity of regrowth 24 h biofilm after 1 min killing treatment. (d) Fluorescence intensity of regrowth 24 h biofilm after 5 min killing treatment.

The resazurin metabolism assay is a high-throughput, simple assay which is typically ideal as an initial screening method in multibiofilm to determine various treatment condition. The absorbance rates of the biofilm on HA disks right after killing and 24 h after killing were established in Figure 3 to assess the impact on acid production. The absorbance rates of high concentration of DMADDM and CHX groups after 1 min (Figure 3(a)) and 5 min (Figure 3(b)) killing treatment were significantly lower than that of the control group (*p *< 0.05). In particular, the biofilm on the HA disks treated with 0.2% CHX after 5 min killing produced the least lactic acid and remained lowest after 24 h regrowth with absorbance rates of 259.59 ± 39.59 and 168.55 ± 34.00, respectively (Figures 3(b) and 3(d)). The high concentration of DMADDM group was significantly different from the control group right after killing (Figure 3(a)) but was not significantly distinct in acid production after 24 h regrowth (Figure 3(c)). The results were comparable to that of the MTT assay (Figure 2) and suggested a promising but short-time inhibition on three-species biofilm with DMADDM and CHX. No significant difference of biofilm metabolism was observed between NaF and control group for 5 min killing (Figures 3(b) and 3(d)).

### 3.4. Biofilm Images Observation

**Figure 4:**
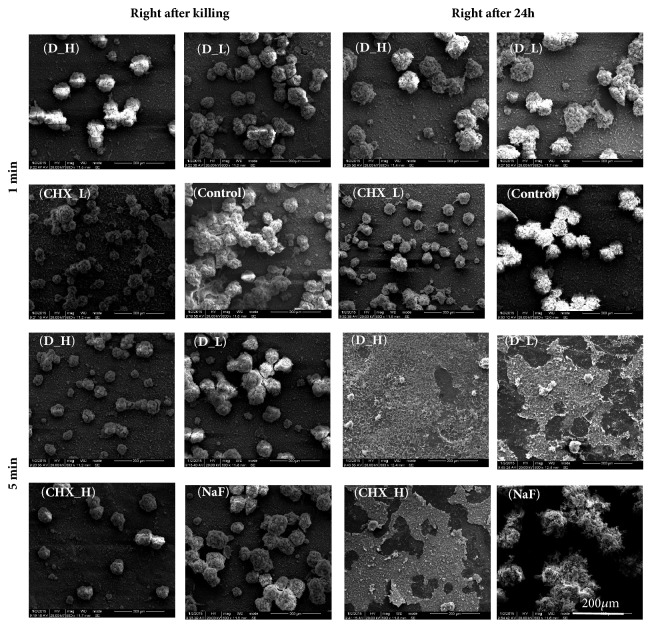
SEM observation of three-species biofilm on hydroxyapatite disk after DMADDM or CHX or NaF treatment.

Figure 4 showed the SEM micrographs of typical biofilm on adhesive disks in different groups at different time points. DMADDM groups and CHX groups inhibited the development of three-species biofilm in varying degrees. Compared to the dense biofilm of control group and NaF group, biofilm on the adhesive disks did not fully develop even after 48 h. While the 200 *μ*g/mL DMADDM group and 0.2% CHX group had the strongest antibacterial activity, only a few bacteria were observed on the surface of the disk.

### 3.5. The Microbial Composition Changes

**Figure 5:**
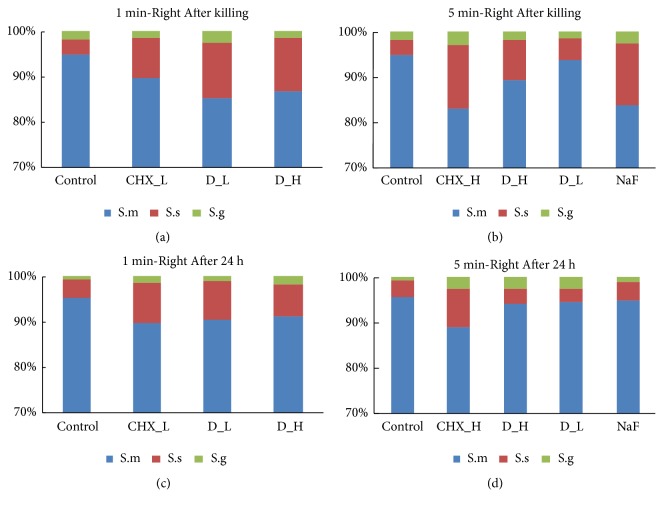
Microbial composition analysis of three-species biofilm on hydroxyapatite disk by TaqMan real-time polymerase chain reaction. (a) Microbial composition analysis after 1 min killing treatment. (b) Microbial composition analysis after 5 min killing treatment. (c) Microbial composition analysis of regrowth 24 h biofilm after 1 min killing treatment. (d) Microbial composition analysis of regrowth 24 h biofilm after 5 min killing treatment.

Figure 5 displayed the ratio variation of three bacteria in three-species biofilm right after killing and 24 h regrowth after killing. Compared to control group, there was a significant decreasing proportion of* S. mutans* in biofilm right after killing treatment, and the proportion of* S. sanguinis* and* S. gordonii *increased accordingly (Figures 5(a) and 5(b)). Similarly, as for 24 h regrowth biofilm after 1 min or 5 min killing treatment, the proportions of* S. mutans* remained decreased trends in DMADDM and CHX groups, compared to control group (Figures 5(c) and 5(d)).

## 4. Discussion

In the present study we investigated the DMADDM-containing solutions with regard to their cytotoxicity on oral cells and antimicrobial effects on oral bacteria, using solution containing CHX and NaF as control group. There existed a certain usage concentration interval with regard to their cytotoxic activities in vitro. The antimicrobial ability observation proved that these solution exerted instant plaque-inhibiting effects, helping control biofilm formation; meanwhile, they could help regulate oral biofilm composition to healthy direction, which suggested that DMADDM exerted great clinical application potentials.

Biocompatibility is very important for all kinds of biomedical agents in clinical applications. The effectiveness of an ideal mouthwash with antimicrobial agent depends on its ability of killing microbes meanwhile causing minimal toxicity to host cells [30]. So in vitro and in vivo potential toxicity of mouthwashes with antibacterial agents should be evaluated before clinical use. But sometimes in vitro studies used to evaluate drug toxicity tended to display cell toxicity, but they are still widely used in clinical treatment under certain conditions. For example, 0.2% CHX is widely used in commercial mouthwash, but 0.2% CHX in the present study exerted obvious toxic effects on human buccal epithelial cells viability, even concentrations higher than 0.2% producing significant cell death [31]. Similarly, in spite of its recognized antimicrobial effect and other beneficial properties, previous in vitro experiment showed that aqueous CHX solutions induced a dose- and time-dependent cells cytotoxicity on odontoblast-like cells [32]. Another common used mouthwash agent, cetylpyridinium chloride (CPC) possessing antimicrobial activity, was also reported to have a negative impact on L929 fibroblast viability [33]. In the present study, we also evaluated the cytotoxic activities of DMADDM at simulate clinical application time (1 min or 5 min). The results indicated that DMADDM above concentration of 40 *μ*g/mL showed significant cytotoxic effects on human buccal epithelial cells. In this case, DMADDM with concentration of less 40 *μ*g/mL should also be acceptable for clinical usage. Above that, the optimum, safe, effective concentration should also be considered with caution in the next preclinical tests.

Mouthwashes containing quaternary ammonium compounds have been reported to be with good plaque-inhibiting effects. For example, CPC, as a cationic quaternary ammonium compound, has been added to some types of mouthwashes [34, 35]. The active ingredient of quaternary ammonium compound has a broad antibacterial spectrum of activity, although its mechanism is complex. In the present study, DMADDM was firstly investigated its antibiofilm ability on oral biofilm after 1 min and 5 min exposure. It was commonly accepted that the cationic agents of QAMs exhibited antibacterial activity by the absorption of positively charged monomer onto the negatively charged cell surfaces of the bacteria [36]. Interestingly, the metabolic activity or acid production ability of biofilm after exposure to 40 *μ*g/mL DMADDM did not decrease. It may be caused that metabolism ability of bacteria was different under different drug pressure. Further research should be conducted, e.g., using long-term flowing cell biofilm models, to evaluate their dynamic antibacterial effects. Similarly, CHX is also a positively charged organic bactericidal agent, which could effectively inactivate bacteria by targeting membrane and cellular components, which is considered the gold standard in the antiseptic treatment of the oral cavity [31]. This is in agreement with the mechanism of action of QAMs, whose antibacterial effects include binding well to bacterial cell membranes, increasing their permeability, initiating leakage, and precipitating intracellular components [37, 38]. The treatment of biofilm with 0.2% CHX was included in the study as control. The antibacterial effect observed by CHX was obvious after being challenged with CHX for 1 min or 5 min, which also occurred in the bacterial renewal/regrowth situation. But NaF did not affect bacteria growth and metabolism, while its main effect is minimizing apatite dissolution, which is also consistent with previous studies [39, 40]. It was reported that NaF offered the best protection when enamel encountering bacterial demineralization [41, 42].

Dental plaque is a biofilm composed of multispecies microbial communities [3, 43], which could cause a variety of biofilm-related oral infections including dental caries, pulp, and periapical diseases [7, 44]. Consequently, eradication of the microorganisms or disrupting the biofilm is one of the primary goals for various oral hygiene approaches [9]. Based on our previous researches [28, 45], multispecies biofilm model was chosen in our present experiment design, because it can better represent and simulate microorganisms in nature, avoiding the limitation of single species biofilm and poor repeatability of saliva-derived microbial biofilm. The model used in the study is of great advantages because of the inclusion of cariogenic* S. mutans* and noncariogenic* S. sanguinis* and* S. gordonii*, which could help evaluate the effects of different antibacterial solutions on biofilm viability and composition. It was reported that* S. mutans* could produce antistreptococcal bacteriocins to suppress* S. sanguinis *and* S. gordonii *[46, 47]; however,* S. sanguinis* and* S. gordonii* could also produce hydrogen peroxide to influence* S. mutans* growth [46, 48]. The initial inoculation amount of the mixed three strains was ensured to be equal with each other in the present study. After exposure to DMADDM for a short time, the proportion of* S. mutans* in biofilm decreased to some extent. Correspondingly, the ratio of* S. sanguinis* and* S. gordonii* in multispecies biofilm increased. Also, the results were in agreement with our previous study in which DMADDM-containing dental materials could decrease* S. mutans* biofilm acidogenicity and regulate the balance of biofilm to healthy tendency [45]. Moreover, bacteria competed with one another for limited resource in biofilm. It was speculated that dual pressure of both competitions between bacteria and antibacterial agent (DMADDM) conditions led to the proportion shift in multispecies biofilm and the change of biofilm development tendency. Taken together, the use of topical DMADDM-containing solutions decreased the ratio of caries associated* S. mutans* in multispecies biofilm to some extent, which revealed its potential benefits to dental caries. However, there existed some limitations about the biofilm model used in our study, e.g., in vitro study, biofilm model with only three species, all belonging to the same genera; thus, in suit salivary biofilm or in vivo study is needed in further works.

## 5. Conclusions

Overall, DMADDM displayed favorable biofilm-inhibiting effects in short time and played a certain role in the aspect of inhibition of subsequent 24 h biofilm growth. Meanwhile, DMADDM could decrease the ratio of cariogenic bacteria (*S. mutans*) among the three-species biofilm. Therefore, DMADDM-containing solutions are promising for more dental applications, e.g., oral mouthwash in the future.

## Data Availability

The data used to support the findings of this study are available from the corresponding author upon request.
